# Microbiomes other than the gut: inflammaging and age-related diseases

**DOI:** 10.1007/s00281-020-00814-z

**Published:** 2020-09-30

**Authors:** Aurelia Santoro, Jiangchao Zhao, Lu Wu, Ciriaco Carru, Elena Biagi, Claudio Franceschi

**Affiliations:** 1 Department of Experimental, Diagnostic and Specialty Medicine (DIMES), Alma Mater Studiorum, University of Bologna, Bologna, Italy; 2 Department of Animal Science, Division of Agriculture, University of Arkansas, Fayetteville, AR 72703 USA; 3 CAS Key Laboratory of Quantitative Engineering Biology, Shenzhen Institute of Synthetic Biology, Shenzhen Institutes of Advanced Technology, Chinese Academy of Sciences, Shenzhen, 518055 China; 4 Department of Biomedical Sciences, University Hospital (AOU) - University of Sassari, Sassari, Italy; 5 Department of Pharmacy and Biotechnology (FABIT), Alma Mater Studiorum, University of Bologna, Bologna, Italy; 6 Laboratory of Systems Medicine of Healthy Aging and Department of Applied Mathematics, Lobachevsky University, Nizhny Novgorod, Russia

**Keywords:** Inflammaging, Microbiota, Evolution, Immunosenescence, Aging

## Abstract

During the course of evolution, bacteria have developed an intimate relationship with humans colonizing specific body sites at the interface with the body exterior and invaginations such as nose, mouth, lung, gut, vagina, genito-urinary tract, and skin and thus constituting an integrated meta-organism. The final result has been a mutual adaptation and functional integration which confers significant advantages to humans and bacteria. The immune system of the host co-evolved with the microbiota to develop complex mechanisms to recognize and destroy invading microbes, while preserving its own bacteria. Composition and diversity of the microbiota change according to development and aging and contribute to humans’ health and fitness by modulating the immune system response and inflammaging and vice versa. In the last decades, we experienced an explosion of studies on the role of gut microbiota in aging, age-related diseases, and longevity; however, less reports are present on the role of the microbiota at different body sites. In this review, we describe the key steps of the co-evolution between *Homo sapiens* and microbiome and how this adaptation can impact on immunosenescence and inflammaging. We briefly summarized the role of gut microbiota in aging and longevity while bringing out the involvement of the other microbiota.

## Introduction: inflammaging and immune system in aging

The lifelong adaptation of the body to the insult from bacterial/viral infections and other stressors represents the origin of a profound age-related remodeling of the immune system (IS) known as “immunosenescence” [1–6] that supports the chronic low-grade inflammatory status called “inflammaging” [6–9]. Even though both immunosenescence and inflammaging (representing two sides of the same coin) may contribute to a higher susceptibility to age-related diseases, several studies demonstrated that they are also necessary to extend survival/longevity [10]. The phenotype of old people and centenarians is indeed surprisingly complex and very dynamic and is the consequence of the ability of the body to respond/adapt to the detrimental stimuli we are exposed to throughout our lifetime [9]. This phenomenon has been conceptualized as “remodeling,” which can be considered a general theory of aging [1, 7]. Centenarians are characterized by high levels of anti-inflammatory molecules [11–14] in the attempt to counteract the increase of inflammaging and find an optimal balance between pro- and anti-inflammatory mechanisms, which likely allowed them to reach the extreme limit of human lifespan [15]. This remodeling is shaped by the immunological history of the organism, a concept dubbed “immunobiography” [16]. According to this idea, everyone has a peculiar immunobiography and consequently a personal inflammaging/immunosenescence. In this scenario longevity, aging and age-related diseases represent a continuum without precise boundaries with the extremes represented by diseases on one side and by centenarians, the best example of successful aging, on the opposite side [17].

Inflammaging can be sustained not only by a variety of external and internal stimuli such as pathogens (non-self) and cell debris and misplaced molecules (self) but also by nutrients and microbiota which are considered “quasi-self” because they come from outside but are tolerated from the IS [18]. In particular, the complex bacterial community that populates different body sites and that represents an evolutionary adapted ecosystem contains an immense diversity of genes that interact directly with human physiology to carry out vital functions [19] and affect the efficiency of the host IS. However, microbiota substantially changes with aging and related disease outcomes [20]. The age-related microbiota changes (dysbiosis) may contribute to inflammaging because long-term stimulation of IS may cause immunosenescence. Such inflammatory condition might make the host more sensitive to potentially dangerous bacteria which in turn contribute to the progression of various pathological conditions in older adults [19].

The present review not only will discuss the co-evolution of microbes and humans and summarize the main findings regarding the gut microbiota (GM) in aging and age-related disease as well as in longevity, but will also focus on the role of many other microbial sites in the human body which are less studied than those in the gut but have a role in healthy and unhealthy aging.

## Humans are metaorganisms: co-evolution between *Homo sapiens* and the microbiomes

Bacteria are older than humans; they were already present on the Earth when eukaryotic cells arose about 2.2 billion years ago. Together with archea, fungi, protists, helminths, and viruses, some bacteria became host-associated and started a long history of co-evolution [21]. Due to symbiotic relationship with the various microbial communities, collectively called “microbiota” present in various anatomical locations of the body, humans have to be considered as a metaorganisms (also termed superorganisms or holobionts) [22]. Trillions of individual bacterial cells colonize the mouth, upper airways, skin, vagina, genito-urinary, and intestinal tract representing a highly integrated ecosystem, which undergoes dynamic changes through time to adapt and respond to environmental signals. The intimate relationships between humans and bacteria have molded the phenotypes in our ancestral lineages. Evidence shows that there is an overlap of the phylogenetic trees of the bacterial microbiota and of primates [23] demonstrating the host-microbiota co-evolution, also genetic [24], and the transmission of microbes within the species through the generations [25].

Environments underwent drastic modifications during human evolution, and climate changes and dietary modification (switch from herbivorous to carnivorous habits and experiences of famine) have been key selective pressures [26]. Mutations, through the natural selection process, point the way to survival and evolutionary adaptation, improving fitness in the new environments. Although the human microbiome offers energy-sparing traits for the human host, several studies exist on the adaptive survival traits to starvation on the human genome, while little is known regarding the microbiome adaptation [25]. Beneficial adaptation to environmental changes will therefore offer advantages to species, and this is the challenge that modern and urban environments are posing to human health.

**Fig. 1:**
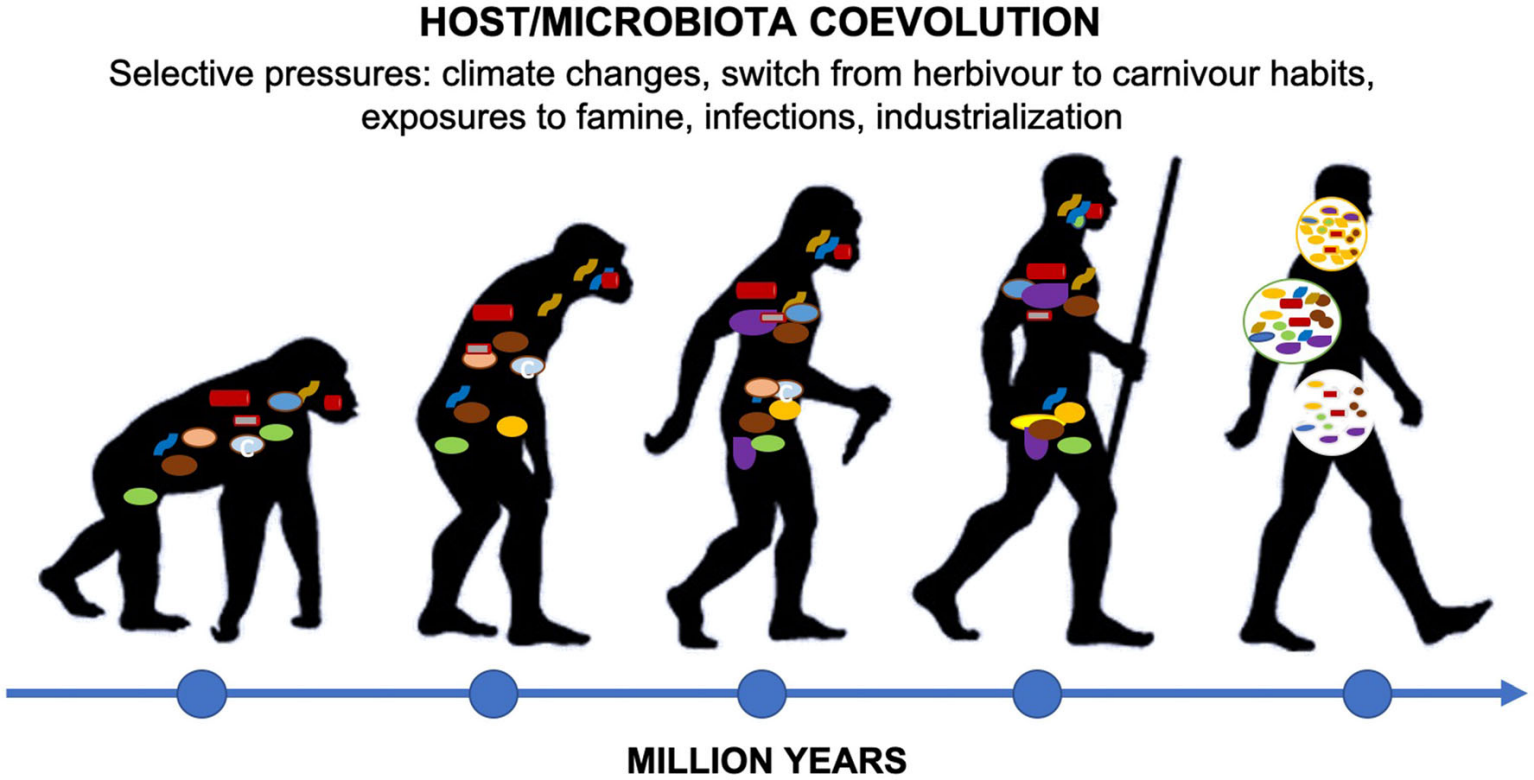
Co-evolution of *Homo sapiens* and microbiota. During human evolution, environments underwent drastical modifications, and climate changes, dietary modification, infections, and industrialization have been major selective pressures [26]. Together, host and microbiota evolved an IS able to prevent the colonization of the interior of human body restricting the microbiota at the interface with the body exterior and invaginations, and host IS developed complex mechanisms to identify and destroy invading microbes, while preserving its own bacteria [25]

Together, the host and microbiota evolved an IS able to restrict bacteria at the interface with body exterior and invaginations while preventing the colonization of internal organs of human body. Host IS recognizes antigens of microorganisms such as DNA, RNA, and cell wall components through toll-like receptors (TLRs) and activates downstream intracellular signaling circuitries to generate immune responses. However, the host IS co-evolved with the microbiota to develop complex mechanisms to recognize and remove invading microbes, while preserving its own bacteria [25] (Fig. 1).

Modern lifestyles are characterized by the transition of traditional foods towards industrial products, use of antibiotics and vaccines, extreme hygiene measures, increase of the rate of cesarean sections, and use of formula instead of breast feeding. These factors according to the “hygiene hypothesis” [27] dramatically impact on microbiota reducing human exposure to microbial symbionts and led to shrinkage of the core microbiome. The progressive disappearance of the functional microbial component (mainly from the gut) of the human IS can contribute to the decrease of its resilience and homeostasis, predisposing individuals to several modern diseases, such as allergy, autoimmune disorders, obesity, inflammatory bowel diseases, and type II diabetes [28–33].

The microbiota associated with the intestinal tract (the GM) is currently the most studied. Microbes in the gut are fundamental for the metabolism of complex vegetal polysaccharides, the biosynthesis of vitamins, and the modulation of fat storage and increase our capacity to extract energy from food [30, 31]; moreover, they are able to strongly control innate and specific immunity. Recent studies on germ-free and gnotobiotic mice revealed that the intense and dynamic crosstalk with the intestinal microbiota strongly impacts the development, education, and homeostasis of the intestinal IS [34]. The GM is associated with various disorders in humans. Alterations in composition, diversity, and metabolites derived from the GM are connected with impairments of different organs of the human body such as brain, lung, joint, heart, liver, and adipose tissue [35]. Evidence for a causative role of the gut bacteria is strongest in metabolic disease [35]. Many independent studies described the microbiome changes as a function of age [36–38], and our research group has shown that centenarians have their peculiar GM [39, 40]. Given that an alteration in GM composition has been linked to different diseases including those age related, the study of GM composition in aging and longevity underwent an explosion in the last decades.

## Summary of the main findings on aging and inflammaging referred to the gut microbiome

The GM undergoes both compositional and functional changes along with physiological modifications that characterize the advancement of age [38]. Such changes, documented by studies performed on populations of different geographic origin, can be summarized in (i) a progressive decrease in butyrate-producing, anti-inflammatory bacterial genera such as *Faecalibacterium* and *Roseburia*, (ii) a decrease in biodiversity, and (iii) an increase in the proportion of otherwise low-abundant and potentially harmful bacteria (i.e., pathobionts), such as members of the families *Enterobacteriaceae*, *Streptococcaceae*, and *Staphylococcaceae* [41–43]. It has been proposed that the increase of pathobionts can be promoted by the low-grade inflammatory status at the level of the intestinal mucosa, which is part of the general process of inflammaging that accompanies the age advancement [44, 45]. Indeed, inflammation is well known to foster the bloom of pathobionts [46] that, in turn, sustain the inflammation by overtaking mutualistic symbionts able to produce short-chain fatty acids (SCFAs), in particular butyrate [47]. SCFA producers play a crucial role in the human gut by promoting immune homeostasis and counteracting inflammation [48]; thus, their progressive decrease can consolidate and nurture inflammatory processes, generating a sort of self-sustaining loop between inflammaging and GM age-related changes. The altered biodiversity often observed in elderly people could contribute to this process by failing to offer an alternative metabolic pathway for SCFA production. In fact, a healthy adult-like microbiota is characterized by a high functional redundancy, sustained by high level of phylogenetic biodiversity, that cope with compositional changes that can occur in response to environmental events [49]. This feature is more likely to lack in the gut ecosystem of elderly people, resulting in insufficient adaptation to environmental and dietary changes and, possibly, diminished ability to produce important metabolites.

In this scenario, it is natural to wonder what comes first, inflammation processes at the mucosal level or microbiome changes, the “chicken-and-egg question.” Being aging an extremely complex and multifactorial process, it is far from easy to provide an answer, also because microbiota itself can be affected by different aging covariates. In fact, age advancement also promotes changes in lifestyle and dietary habits, in response to physiological changes in thresholds for taste and smell, decreased physical activity, masticatory dysfunctions, etc. For this reason, the diet of elderly people may include less fibers and proteins and a decreased introduction of uncooked, fresh food [50], with negative effects on microbiota diversity and on abundance of fiber degrading and SCFA-producing bacteria. A recent paper showed that undergoing Mediterranean diet for 1-year changes gut microbiota composition of elderly improving health status and reducing frailty [51].

Aging comorbidities, such as frailty, diabetes, cardiovascular diseases, as well as cancer, can enhance the age-related changes in GM that, in turn, can promote their consolidation or speed up their progression [45, 52–54]. For instance, immunosenescence can result in inappropriate response towards symbiotic microbiota components and/or decreased capability to control pathogen invasion, contributing to chronic inflammation and, on the long term, to the onset of some cancer types, i.e., colorectal cancer [55, 56]. The decrease of biodiversity could also represent a weakening factor for the host defenses against pathogen invasion, for instance promoting the onset of infections by opportunistic bacteria such as *Clostridium difficile*. *C. difficile*-associated diarrhea is a major nosocomial complication for frequently hospitalized elderly [57]. Finally, very recently, a field of particular interest for microbiome and aging research is the possible association between inflammatory and debilitating diseases such as physical frailty, sarcopenia, and osteoarthritis [58] with GM. Even if a direct and causal link between microbes and these frequent age-related conditions has yet to be explored, available data on adults provide muscle mass and function as well as bone and joint [58–61] that could become of importance in future intervention strategies, including diet, supplements, and probiotics/prebiotics, to increase the chances to achieve a “healthy aging” [61, 62].

## In addition to the gut microbiome, which role do the other microbiomes play in aging and age-related diseases?

The ecosystem of the human gut is the most studied microbiome because of its pervasive role due to its capacity to convert environmental signals and dietary nutrients in bioactive compounds which signal to distant organs and tissues in the body. Gut bacteria are thus able to connect to the immune and hormone system, to host metabolism, to the central nervous system as well as other functions of the host [35, 63].

However, beyond GM, all the other microbiomes present in the different human body sites (Table 1) contribute to host physiology, and may also play a critical role in host specific pathological conditions, fuelling inflammaging and contributing to immunosenescence, when the microbial equilibrium is altered as a consequence of external/internal detrimental stimuli.

**Table 1:** Predominat bacteria in specific body site

	Predominant phyla	Reference
Body site		
Mouth	*Firmicutes* (e.g., *Streptococcus*); *Bacteroidetes* (e.g., *Prevotella*); *Proteobacteria* (e.g., *Haemophilus*); *Actinobacteria* (e.g., *Actinomyces*); *Spirochaetes* (e.g., *Treponema*); *Fusobacteria* (e.g., *Fusobacterium*)	[154–156]
Nose	*Actinobacteria, Bacteroidetes, Firmicutes, Proteobacteria*	[157]
Lung	*Proteobacteria*, *Firmicutes*, *Bacteroidetes* and *Actinobacteria*	[80]
Skin	*Actinobacteria* (Propionibacterium, Corynebacterium) and *Firmicutes* (Staphylococcus)	[89, 90]
Gut	*Firmicutes* (e.g., *Lachnospiraceae, Ruminococcacea*e), *Bacteroidetes* (e.g., *Bacteroides* and/or *Prevotella*, depending on ethnicity), *Actinobacteria* (*Bifidobacterium*, in different abundance according to the host’s age)	[37, 41, 42]
Vagina	*Firmicutes* (*Lactobacillus*)	[104, 107, 110]
Genito-urinary tract	*Actinobacteria* (*Gardnerella, Corynebacterium*) and *Firmicutes* (*Lactobacillus, Streptococcus*)	[128–131, 141]

Each body habitat has indeed a unique configuration of bacterial microbiota that reflects properties of the local environment and changes with age shaping host development and vice versa. Within each habitat, there is large variation between individuals; however, the compositional oscillations in an individual’s microbiome over time are less abundant than inter-individual alterations at a particular stage of life [55, 64].

Although relatively few studies are present on the role of the different microbiomes in aging and pathologies, the following sections will describe the main findings regarding the aging of the microbiome of other human ecological niches such as oral cavity, lung, skin, vagina, and genito-urinary tract and the development of clinical diseases that are common among older adults such as pneumonia and chronic obstructive pulmonary disease (COPD), urinary tract infection, reactive airways disease, and other malignancies.

### Oral and nasal microbiome in elderly

The microbiota of the oral cavity is extremely diverse containing as many as 700 or more species [65–68], of which the vast majority belong to the phyla *Firmicutes*, *Actinobacteria*, *Bacteroidetes*, *Proteobacteria*, *Fusobacteria* [68–70], and *Spirochaetes* [68]. Bacterial colonization in the oral cavity and oropharynx occurs mainly on the lips, teeth, cheeks, subgingival and supragingival surfaces, hard and soft palate, and tonsils [68]. The Human Microbiome Project sampled many of these locations and found that, in most, the dominant genera were *Streptococcus*, followed by *Haemophilus*, *Actinomyces*, and *Prevotella* in the buccal mucosa (cheek), supragingival, and subgingival plaque, respectively [66]. It is thought that there is a “core microbiome” present in the majority of individuals which consists of *Actinomyces*, *Atopobium*, *Corynebacterium*, *Rothia*, *Bergeyella*, *Capnocytophaga*, *Prevotella*, *Granulicatella*, *Streptococcus*, *Veillonella*, *Campylobacter*, *Cardiobacterium*, *Haemophilus*, *Neisseria*, TM7, and *Fusobacteria* [68]*.* Compared with the gut, the relationship between the oral microbiome and aging is not as well studied [65]. Ogawa et al. analyzed the oral microbiome of elderly individuals living in a nursing home (EN) and those that live independently (control) and found the EN group was less diverse at the phyla level but not at the genus level. The EN group had a higher relative abundance of *Actinomyces*, *Streptococcus*, *Bacilli*, *Selenomonas*, *Veillonella*, *Haemophilus*, and a lower relative abundance of *Prevotella*, *Leptotrichia*, *Campylobacter*, and *Fusobacterium* compared with the controls [71]. Furthermore, Singh et al. analyzed oral microbiomes of healthy aging (HA) and non-healthy aging (NHA) individuals and found that HA had a higher alpha diversity than NHA. The only genus that was more abundant in HA of the overall most abundant genera was Neisseria. Haemophilus, Fusobacterium, and Capnocytophaga were all increased in HA, but were lower abundance genera. [72].

Inflammaging likely plays a role in the relationship between aging and oral microbiome [73]. During aging, many changes occur in the oral cavity that can lead to chronic inflammation, which can increase an individual’s susceptibility to oral disease [74]. Additionally, age is considered a risk factor for oral diseases, such as periodontal disease, which, in the USA affects around 60% of adult population [73]. Although a causative role has not been demonstrated yet, it is interesting to mention that periodontal pathology has been associated with atherosclerosis, suggesting that bacteria from the oral cavity may play a role in the onset of atherosclerosis and cardiovascular disease [75]. Feres et al. analyzed the subgingival microbiota within different age groups and found that in healthy individuals, there were no differences in the amounts of the analyzed taxa within the age groups but noted that the older adults (> 64) trended towards an increased abundance of three *F. nucleatum* ssp. However, in individuals with periodontal disease, the younger group (< 35) had an increased amount of the *P. gingivalis* and *T. forsythia* compared with the older groups. Furthermore, the oldest group had higher levels of four Actinomyces, especially Actinomyces naeslundii and Actinomyces oris [73].

While much is known about the nasal microbiome early in life, there is less research over the nasal microbiome in elderly individuals. Bomar et al. stated that one study found in elderly individuals the nasal microbiota has a high abundance of *Streptococcus*, and a study analyzing the nasal microbiota of elderly individuals with Parkinson’s disease (PD) found that in both PD and healthy controls, the composition of the nasal microbiota resembled that of the middle-aged adult. An additional study analyzing the nasal microbiota of elderly individuals found no difference in diversity between those living in a nursing home and those living independently. However, the relative abundance of *Lactobacillus reuteri*, *Streptococcus*, *Staphylococcus epidermidis*, and *Rothia mucilaginosa* were increased in individuals residing in the nursing home [76]. Koskinen et al. examined the relationship between the nasal microbiota and olfactory function. They found that *Faecalibacterium* and *Porphyromonas* strongly correlated with a reduction of olfactory function, and *Corynebacterium* members correlated with a reduction in odor discrimination and threshold. Interestingly, they also found that compared with the normal threshold scores, the individuals with lower scores had a more diverse microbiome [77]. Additionally, Rullo et al. characterized both the oral and nasal microbiome in newly diagnosed neovascular age-related macular degeneration (AMD) and healthy controls. In the oral microbiome, *Propionibacteriales*, *Rothia*, *Staphylococcus*, and *Cornyebacteriaceae* were increased in AMD, while *Fusobacterium* and Bacilli were higher in controls. In the nasal microbiome, *Actinomycetaceae*, *Gemella*, Proteobacteria, Actinomyces, and Veillonella were significantly higher in AMD, and when compared with controls, *Streptococcus* underwent the largest relative shift in AMD. Although not relatively abundant, Burkholderiales were also significantly increased in AMD, while Clostridia were increased in the control group [78]. More studies are desired to elucidate the relationship between the oral and nasal microbiome and aging in order to prevent upper respiratory tract infection [76] and subsequent lower respiratory tract, such as lung (which is covered in the next section) infections.

### Lung microbiome

More and more evidence show that there are also diverse resident microbes in healthy lungs. The main phyla are Proteobacteria, Firmicutes, Bacteroidetes, and Actinobacteria [79]. The microbiome of the lung tissue is still largely unknown, and many studies have linked changes in the lung microbiome to the development of chronic lung diseases, such as cystic fibrosis (CF) or chronic obstructive pulmonary disease (COPD). However, the complex relationship between lung microbiota and disease remains to be elucidated. The surface of healthy lung is a dynamic environment, and debris and microorganisms from the mouth and nose continue to enter this respiratory organ, and the ciliary cells of the bronchus can remove these debris and invading bacteria through rhythmic movement.

In general, the lung microbiota is diverse, and it varies greatly among specific individuals and is dominated by 9 core genera: *Prevotella*, *Sphingomonas*, *Pseudomonas*, *Acinetobacter*, *Clostridium*, *Megasphaera*, *Veillonella*, *Staphylococcus*, and *Streptococcus* [80]; some of them such as *Prevotella*, *Veillonella*, and *Streptococcus* were also frequently observed in the oral cavity [81]. Although the respiratory system and its mucosa are interconnected, the lung microbiota has very distinctive features that set it apart from the upper respiratory tract [82].

Studies have shown several factors that are related to changes in lung microbiology, such as air pollutions, smoking, aging, and diseases. For instance, air pollution caused an increase in the relative abundance of potentially pathogenic bacterial groups such as *Streptococcus* and *Neisseria* [83]. The characteristics of the lung microbiome also change with the natural process of aging. Evidence has shown the loss of diversity of lung microbiota with increasing age and lung disease severity [79, 84], and antibiotic exposure was strongly associated. Aging-related immune dysfunction also affects the lung microbiome [84, 85]. A recent analysis of the lung microbiota of 167 severe asthma patients also revealed significant differences among patients with different inflammatory phenotypes [86].

### Skin microbiome and aging

The human skin is inhabited by a large and diverse community of both bacteria and fungi that contributes to the protection against invading pathogens and educates the IS [87, 88]. The composition of the skin microbiome primarily depends on the physiology of the skin site: for instance, sebaceous sites are usually dominated by *Propionibacterium* members, whereas moist areas, such as feet and elbow’s bend, are usually populated by *Staphylococcus* and *Corynebacterium* [89, 90]. This is related to the fact that microbes inhabiting skin are selected based on their ability to utilize resources present in sweat, sebum, and/or the debris of dead skin cells present in the outermost layer of human epidermis [88].

Being that the skin is such an exposed environment, it would be natural to think of its microbiome composition as much less stable than that of more protected environments such as gut or vaginal ecosystem; conversely, it has been proven by longitudinal studies that skin microbiome composition is stable, especially in sebaceous sites, e.g., the forehead [91]. Studies on individuals of different ages have provided evidences that human skin microbiome stabilizes its compositional structure around the age of 3 years, similarly to what happens for the GM [92], but it undergoes a dramatic restructuring at the time of puberty, when changes in hormone concentration stimulate sebum production [93]. Age-related changes in skin microbiome are interesting because of the many skin disorders associated with the puberty transition as well as the different propensity for atopy shown by the children and adult’s skin [88].

Analogously to pubescent individuals, skin structure and physiology change for elderly along with aging-related endogenous intrinsic factors, e.g., changes in cellular metabolisms, immunosenescence, and altered hormone condition [94]. Changes in skin structure also depend upon lifestyle choices and environmental challenges taken during the whole adult life, including cumulative UV exposure, smoking, and pollution [95]. These factors together usually determine a decrease in sweat, sebum, and immune homeostasis, resulting in alterations in skin physiology (e.g., pH, lipid composition). These physiological changes ultimately provide alterations in the microenvironment that affect the skin microbiome composition, especially in relation to the decrease in sebum production [90] and the occurrence of skin ulcers in bedridden elderly [96]. However, the literature focusing on the skin microbiome in elderly population is far from comprehensive, with few studies on limited populations currently available [97–99]. Ying and colleagues (2015) focused on rural and urban populations from Shanghai area (China) and provided evidences that, even if aging had an effect on skin microbiome composition, the rural/urban environment was the most relevant driver for this exposed human microbiome. Shibagaki and colleagues, on the contrary, focused on a small population of healthy Japanese women: besides identified a number of bacterial species that showed differential abundances between older and younger women, the authors provided evidences, confirmed later on by Wu and colleagues that age-related alterations in skin microbiome are site dependent. In forehead, cheek, and forearm, the author found an age-associated decline in *Propionibacterium* abundance, proposedly related to the decrease in sebaceous gland activity which is typical of older age. Indeed, Wu et al. confirmed not only in Sardinian elderly (Italy) but also in centenarians from the same area that *Propionibacterium* in forehead and palm microbiomes decreases along with the advancement of age, with other genera (i.e., *Prevotella*, *Rothia*, and *Veillonella*) becoming overrepresented. Wu et al. also took into account the eukaryotic skin population alongside the bacterial one: skin fungi population seemed to be less affected by the advancement of age, with Malassezia consistently dominating the various skin sites. On the contrary, it has been demonstrated that aging significantly affects the proportion of the abundance of the Archaea counterpart of the skin microbiome, which increases in older age in relation to the lower sebum levels and reduced moisture [100].

More studies, on larger and more geographically spread populations, will offer a comprehensive view of the microbiome changes that occur along with the aging process on the human skin, ultimately providing useful and exploitable information in the field of treating and preventing age-related skin disorders. For instance, elderly is a subgroup of patients with distinct atopic dermatitis manifestation with respect to atopic infants, children, and adults [101], with the atopic problem possibly opening the way to bacterial skin infections [102]. Since it is known that skin microbiome, and in particular an increase colonization by *Staphylococcus aureus*, contributes to the exacerbation of atopic dermatitis [103], it is necessary to understand if and how age-related modification in the skin microbiome, in the different sites, can favor a skin microecosystem in which atopic manifestations, as well as their infectious consequences, are promoted.

### Vaginal microbiome in aging women

Vaginal microbiome is probably the most studied human microbial ecosystem after the gut, because of its well-known relationship with the women health status [104]. Consistently across the whole literature, a vaginal environment dominated by *Lactobacillus* species is associated with vaginal health, with this group of bacteria being considered as keystone for the ecological balance of the vaginal environment. *Lactobacillus* species are responsible for the production of metabolites such as lactic acid and hydrogen peroxide, which contribute to the maintenance of the healthy value of vaginal pH, as well as the creation of a microenvironment in which colonization by anaerobic and microaerophilic pathogens is prevented [105–107]. The recent extensive application of NGS to vaginal samples across different population and physiological conditions allowed for the categorization of vaginal microbiome into a discreet number (5 to 8) of community state type (CST), characterized by different degree of dominance of different *Lactobacillus* species (i.e., *L. iners*, *L. crispatus*, *L. gasseri*, *L. jensenii*) or by the absence of such dominance. CSTs deprived of a strong *Lactobacillus* dominance and enriched in other, often anaerobic, bacteria (e.g., *Streptococcus*, *Atopobium*, *Megasphaera*, *Prevotell*a) were associated to a higher probability of disease or poor health outcome, such as bacterial vaginosis and pre-term delivery [108–111]. In spite of these few possible configurations, the vaginal ecosystem strikes as particularly dynamic and undergoes compositional and functional changes along the woman life, in relation to hormonal changes, the most evident being puberty, pregnancies, and the beginning of menopause. Smoking, diet, hygiene, and sexual practices add complexity to the description of the vaginal microbiome dynamics [104, 112]. While puberty is known to represent the moment of the most dramatic changes in the vaginal microenvironment [112], pregnancy is of outmost interest because of the proven contribution of vaginal microbiome to pre-term labor and delivery [110, 111]; menopause-related changes in vaginal ecosystem have started to interest research only during the last decade [113–118].

Menopause causes modifications of the vaginal environment that include decrease of the mucus layer width, estrogen level, and glycogen production. Such physiological changes are accompanied by modifications in the resident microbiome that includes a depletion in the proportion of *Lactobacillus* members, as well as a general decrease in the absolute number of colonizing bacteria and, consequently, an increase in biodiversity and vaginal pH [112, 118]. The focus of the majority of the research in this field has been how these microbiome changes are connected with genitourinary symptoms that are experienced by a large number of aging post-menopausal women. Indeed, vulvovaginal atrophy (VVA) and genitourinary symptoms of menopause (GSM, including burning, dryness, irritation, and so on) are experienced by approximately half of western post-menopausal women [112, 119]. It was reported that post-menopausal women with none to mild symptoms had significantly higher *Lactobacillus* predominance, and consequently lower biodiversity, than those complaining of more severe vaginal symptoms, whose samples were found enriched in bacteria such as *Prevotella*, *Porphyromonas*, *Peptoniphilus*, and *Bacillus* [114]. Later on, Brotman and colleagues confirmed that post-menopausal women with the most severe VVA showed a vaginal CST belonging to the group IVA, i.e., the one without Lactobacillus dominance and enriched in *Anaerococcus*, *Peptoniphilus*, *Prevotella*, and *Streptococcus*. Even if such studies did not provide answer to the chicken-and-egg question (VVA and GSM-related changes in microbiome cause or are caused by menopausal symptoms?), it was a natural evolution of the research field to wonder if therapies improving menopause-associated disturbances also had some effect on vaginal microbiome. Indeed, several studies showed that hormone replacement therapy, besides being effective in reducing menopausal symptoms [120, 121], influences the vaginal microbiome in a positive manner, by increasing the *Lactobacillus* amount and favoring the re-establishment of a vaginal microenvironment more similar to the one found in pre-menopausal women, i.e., higher glycogen production and lower pH [112, 117, 118]. Based on this, even if the relationship between estrogen level and vaginal microbiome in menopausal women has yet to be thoroughly explored, it is suggested that the maintenance of a vaginal microbiome dominated by *Lactobacillus* is relevant for ensuring a good quality of life for post-menopausal women. For this reason, the possibility of probiotic usage, orally or locally administered, during the management of VVA in aging women has been proposed and explored [122, 123]. A few studies highlighted that *Lactobacillus*-based products have the ability to increase the *Lactobacillus* dominance in post-menopausal women [124–126].

### Genito-urinary tract microbiome and aging

Advances in our understanding of human microbiota especially GM and host interaction has stimulated our interest in other mucosal sites such as the genito-urinary tract microbiome (GUTM). Bladder and lower urinary tract were misunderstood as sterile for a long time. Although in normal physiological conditions, the commensals in GUTM were less abundant but highly variable compared with that in the gut [127], there are several genera commonly observed, such as the most dominant genera *Lactobacillus* and *Gardnerella* in healthy female cohorts [128, 129] and *Lactobacillus* and *Streptococcus* in healthy male cohorts [130, 131]. However, there are not always consistent results among studies for the commensals in the GUTM, which may be caused by the different types of samples and detection methodology [132]. Despite significant differences in gross anatomy and physiology of the lower urinary tract for females and males [133], the urine microbiota in the male and female are dispersed clustered into several “urotypes” rather than have a clearly separated clustering [134]. It is worth noting that the diversity of urine microbiota in healthy men is larger than that of healthy women [134]. Although the urine microbiota may have linkage with vaginal microbiota and seminal microbiota (which was well summarized in review) [135], it was not strongly influenced by the distal regions of the urogenital tract. Whereas a significant overlap between the bladder and vaginal microbiota was identified in a recent study [127], which reveals an interconnected GUTM. Currently, there is no direct comparison between the bladder microbiota (or clean-catch urine microbiota) and seminal microbiota, while comparison of the bacterial communities in semen with those of first catch urine did demonstrate that these specimens shared one-third of species [136]. Till now, there remain some doubts on whether different urogenital sites harbor a unique microbiome.

Many clinical situations such as urinary tract infection [137], interstitial cystitis [138], urinary incontinence [129], the formation of kidney stones (urolithiasis) [139], and even genito-urinary tract cancer such as bladder cancer [140], prostate cancer [141], and kidney cancer [142] have some correlations with the altered GUTM. Those clinical conditions are more relevant to the aging population, for example, the high incidence of urinary tract infection was 40% of men and 28% of women in their 70s [143, 144]. During the aging process, host physiological and lifestyle changes, for instance, the IS function declining, sexual activity frequencies decrease, more frequent medication exposure, may affect the GUTM. There are studies that demonstrated the age-related urine microbiota variations in female, similar with the GM which also showed age-related variations during aging [36, 145, 146]. A study that characterized the urinary microbiota in elderly (average 71.8 years) and younger females in China (average 50.0 years) has found significant differences between them [147]. The relative abundance of *Lactobacillus* and *Bifidobacteria* was negatively related to age, while *Peptococcus* was positively related to age. Moreover, the correlation between a higher level of *Lactobacillus* and diabetes was identified in the elderly, and lower levels of *Peptoniphilus* and *Dialister* were correlated with asymptomatic bacteriuria. While another study in the UK did not find significant correlations between age and diversity of the bladder microbiome in healthy females [148], the *lactobacillus* was observed related to the pre-menopausal females, and *Mobiluncus* was related with post-menopausal females. A study focused on the urine microbiota in the urinary incontinence female and control has found that urine microbiota formed six community types (urotype), which was not significantly associated with the urinary incontinence but was age-related [129]. The young females (< 51 years) have a higher proportion of individuals with *Lactobacillus*-dominated urotype structure (with relative abundance of Lactobacillus > 89%). Interestingly, the younger females without the *Lactobacillus*-dominated urotype were correlated with a significantly high incidence of urinary incontinence but not for the older females. Another similar study that surveyed the urine microbiota only in the urinary incontinence female showed that the younger females (average 55.8 years) have a higher incidence of being positively detected with urine microbes compared with the older female (average 61.3 years), and the *Enterobacteriaceae*-dominant urotypes were detected within females with an average age of 70 years compared with the *Lactobacillu*s-dominated or *Gardnerella*-dominant urotypes with an average age around 54 years [149]. A more recent study describes the less disperse cluster of bladder microbiota in younger women (average 51 years) when compared with the older female (average 59 years) [150]. *Enterobacteriaceae* and other potential pathogens including *Pseudomonas* and *Staphylococcus* are consistent with clinical observations that the older female has an upward trend of getting urinary tract infection [151]. A study that used 16S rRNA qPCR and cultivation methods indicated that the “detectable” urine bacteria are not significantly associated with age; however, *Jonquetella*, *Parvimonas*, *Proteiniphilum*, and *Saccharofermentans* were found enriched in aged individuals over 70 years [152]. Furthermore, an association has been noted between male age and seminal bacteria; the presence of environmental bacteria such as *Pseudomonas*, *Janthinobacterium*, *Gillisia*, *Flavobacterium*, and *Acidovorax* was associated with older age instead of vaginal bacteria such as *L. crispatus*, *L. iners*, *G. vaginalis*, *Dialister*, *Atopobium vaginae*, and *Mobiluncus curtisii* that seemed to be associated with younger age [153]. Although there is still no comprehensive study of the aging-related urine microbiota, the impact of the microbiota on genito-urinary tract homeostasis and disease development is emerging. Future studies will improve our understanding of the causative relationship between the defined microbes and genito-urinary tract diseases. And undoubtedly, age should be considered when we try to use the urine microbiota as a predictor of disease or treatment.

A summary of the findings on the association of aging with changes in microbiomes from the different body sites described in this review is reported in Table 2.

**Table 2:** Summary of the findings on the association of aging with changes in microbiomes from the different body sites

Body sites	Predominant phyla	Cohort location	Recruited subjects number (age range)			Sequencing stage		*α* Diversity (taxonomic)		*β* Diversity (taxonomic)	Aging-related enrichment taxa	Aging-related decreased taxa	Reference
			Young	Elderly	Longevity			Shannon index	Richness	variation among individuals			
Gut	*Firmicutes*, *Bacteroidetes*, *Actinobacteria*, *Proteobacteria*	Sardinia (Italy)	19 (21–33)	23 (68–88)	19 (99–107)	Illumina Hiseq and Miseq	Shotgun metagenomic sequencing and 16S rRNA V3V4	≈	≈	↑	Methanobrevibacter smithii, Bifidobacterium adolescentis	Faecalibacterium prausnitzii, Eubacterium rectale	[145]
	Firmicutes, Bacteroidetes	Bologna (Italy)	20 (25–40)	43 (59–78)	21 (99–104)	Phylogentic microarry and qPCR	16S rRNA	↓		↓	Anaerotruncus, Oscillospira, Christensenellaceae, Eggerthella	Faecalibacterium, Roseburia, Coprococcus	[146]
Skin	Actinobacteria	Sardinia (Italy)	19 (21–33)	23 (68–88)	19 (99–107)	Illumina Hiseq and Miseq	Shotgun metagenomic sequencing and 16S rRNA V3V4	Site-dependent alterations		↑	Staphylococcus, Streptococcus	Propionibacterium	[99]
	Actinobacteria	Shangai (China), urban and rural	24 (25–35)	22 (50–60)		454 Pyrosequencing	16S rRNA	≈	↓				[97]
	Actinobacteria	Japan	18 (23–37)	19 (60–76)		454 Pyrosequencing	16S rRNA	↑	↑		Corynebacterium, Acinetobacter	Propionibacterium, Staphylococcus	[98]
Mouth	Firmicutes (EN), Bacteroidetes (HC)	Osaka, Japan	16 (79–94)	15 (68–101)		Pyrosequencing	16S	*↓ (EN)*			Actinomyces, Streptococcus, Bacilli, Selenomonas, Veillonella, Haemophilus (increased in EN)	Prevotella, Leptotrichia, Campylobacter, and Fusobacterium (decreased in EN)	[71]
		Cambridge, USA/São Paulo, Brazil	periodontis: young 152 (28.5 ± 4.7), middle 833 (35–64); periodontally healthy: young 119 (29.24 ± 6.2), middle 112 (35–64)	periodontitis 99 (69.6 ± 4.2); periodontally healthy 15 (67.8 ± 9.8)		Checkerboard DNA–DNA hybridization					Actinomyces (increase d in elderly with periodontal)	P. gingivalis and T. forsythia (increased in younger with periodontal)	[73]
	Firmicutes	Danbury, CT, USA	33 (70–82)	32 (70–82)		illumina Miseq	16S	↑(healthy aging)			Streptococcus, Veillonella, and Rothia (NHA)	Neisseria, Haemophilus, Fusobacterium, and Capnocytophaga are enriched in healthy aging.	[72]
	Actinobacteria (in AMD)	Ontario, Canada	5 (<59–89)	13 (60>89)		Illumina NextSeq500	16S				Propionibacteriales, Rothia, Staphylococcus, and Cornyebacteriaceae (increased in AMD)	Fusobacterium and Bacilli (higher in controls)	[78]
Nose		Ontario, Canada	5 (<59–89)	13 (60>89)		Illumina NextSeq500	16S				Actinomycetaceae, Gemella, Proteobacteria, Actinomyces, Veillonella, Burkholderiales (significantly higher in AMD)	Clostridia (increased in the control group)	[78]
Lung (sputum)	Firmicutes, Proteobacteria, Bacteroidetes, Actinobacteria	Singapore	24 (22–39)	24 (60–71)		MiSeq Illumina	16S	Not significant different	Not significant different	Not significant different	Firmicutes	Proteobacteria (Haemophilus, Lautropia)	[85]
Bladder (urine)	Proteobacteria, Firmicutes	Zhejiang, China	50 (50.06 ± 7.51)	50 (71.86 ± 6.70)		MiSeq Illumina	16S V3V4	↓ (Not significant)	↓ (Not significant)		Peptococcus	Lactobacillus, Bifidobacteria	[134]
	Firmicutes	UK	23 (20–29)	14 (50–59)		Eurofins Genomics	16S	Not significant different	Not significant different		Mobiluncus	lactobacillus	[148]
	Firmicutes, Proteobacteria	Bristol, UK	13 (20–49)	20 (50–70+)		454 Pyrosequencing	16SV1V3	Not significant different	Not significant different		Jonquetella, Parvimonas, Proteiniphilum, and Saccharofermentans.		[152]

Vaginal ecosystem is not described in this table because the focus of studies concerning this ecosystem is never the aging per se, but the menopause and associated symptoms, therapies, or conditions

## Conclusions and perspectives

**Fig. 2:**
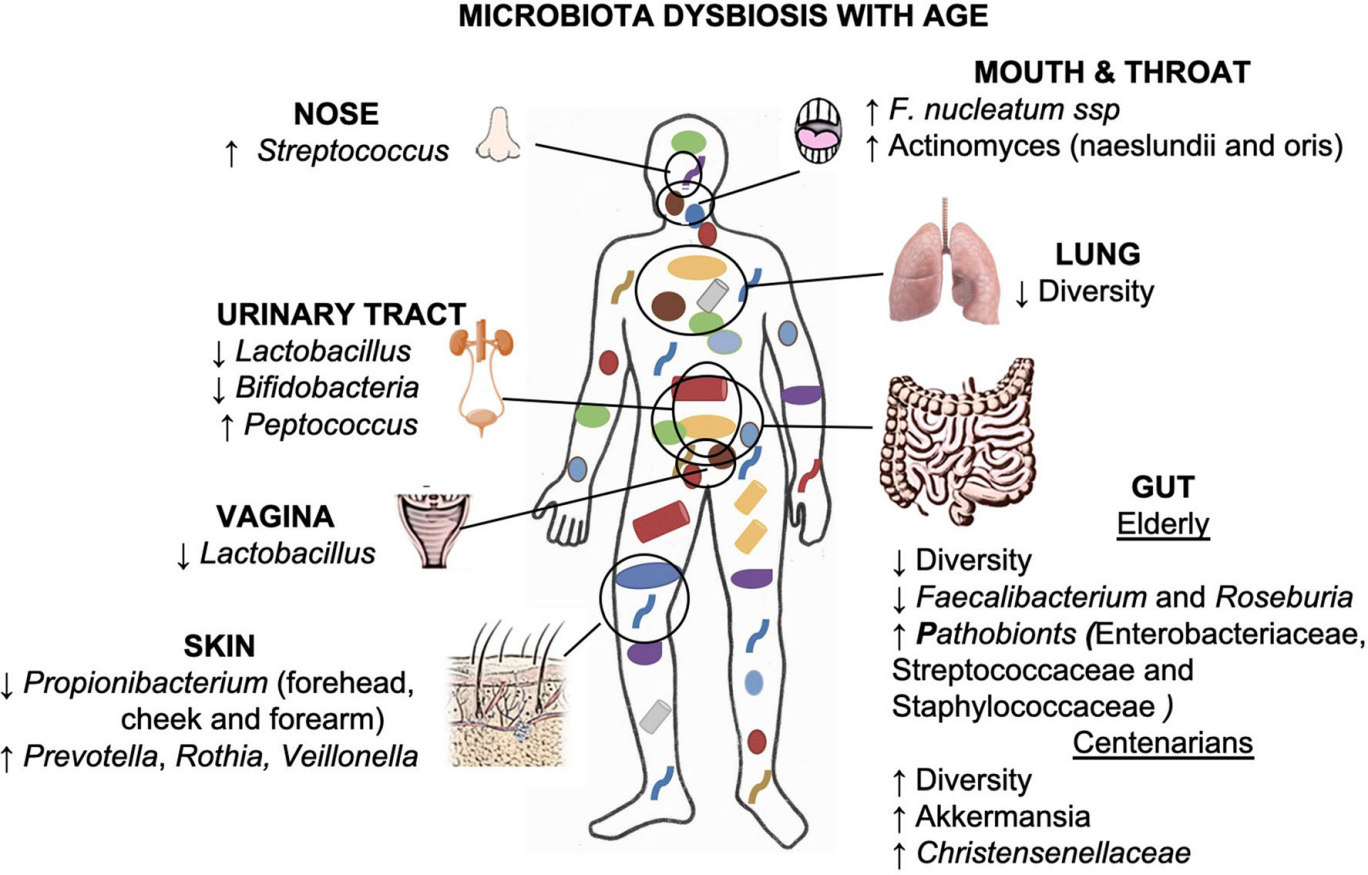
The age-related microbiota changes (dysbiosis) at each body site. Dysbiosis may contribute to inflammaging because long-term stimulation of IS may cause immunosenescence. Such inflammatory state might make the host more sensitive to bacteria, and in turn, alterations in the composition of microbiota are associated with the progression of various pathological conditions in older adults [19]

The human body and its microbiome represent an integrated meta-organism, which results from million years of reciprocal adaptation and functional integration conferring significant advantages for both parties. All the members of this human microbiota participate in host physiology and change according to development and late in the life contributing to health and fitness. The human IS is influenced by the microbiota assembly, composition, diversity, and dynamics, and the interaction of all these features plausibly contributes to the process of inflammaging (Fig. 2). In the last decades, we experienced an explosion of studies on the role of GM in health and disease and the relationship between GM and the other organs and tissues also due to an improvement of the sequencing methods that can be applied to the study of microbiota.

Though the knowledge on human holobiont is increasing as a consequence of the improvement in the assessment of both correlation and causal relationships of the collective microbiome and host functions in health and disease, the complex relationship between humans and the trillions of bacterial cells that form our microbiome remains largely unexplored. The consequences for medicine are challenging, since it is likely that our multifaceted symbiosis affects each aspect of health. Manipulating the intestinal microbiota and microbiome may be helpful for preserving health and treating disease, particularly among older adults. On the contrary, the relationship between the microbiome of other human ecological niches (i.e., oral cavity, lung, skin, vagina, and genito-urinary tract) and the progress of other clinical diseases that are common among older adults remains an important area of future studies. It is also necessary to consider how biological age (assessed by health status and life expectancy) shapes the microbiota and IS and vice versa. Moreover, the complexity of the interactions within the microbiome of the different body sites and between microbes and hosts presents a major challenge; a more concerted and predictive theoretical framework is imperative to progress.

Efforts to standardize specimen preparation and analytical protocols and to increase the availability of the growing body of data should be increased. These technical efforts as well as robust clinical research will improve characterization of the variation in the global human microbiomes, functions of redundancy, disease biomarkers, immigration, effect of lifestyles, and trajectories of development, all of which will establish the basis to understand the progression from health to disease and to efficiently discover new preventive strategies and therapies.
